# Modern Obesity Treatment: Efficacy, Safety, and Future Directions of GLP-1 (Glucagon-Like Peptide-1) and GIP (Glucose-Dependent Insulinotropic Polypeptide) Receptor Agonists

**DOI:** 10.7759/cureus.116245

**Published:** 2026-09-14

**Authors:** Dorota Bartkowiak, Barlomiej Jakubczyk, Adam Lasota, Lukasz Kopoczynski, Michal Wiesner, Lukasz Wodzinski, Kamil Boguszewski, Hubert Porazinski, Agnieszka Młynarska, Oskar Ratajski

**Affiliations:** 1 Medicine, Dr Jan Biziel University Hospital No. 2 in Bydgoszcz, Bydgoszcz, POL; 2 Medicine, Antoni Jurasz University Hospital No. 1 in Bydgoszcz, Bydgoszcz, POL; 3 Cardiology, Samodzielny Publiczny Szpital Wojewódzki im. Papieża Jana Pawła II, Zamość, POL; 4 Medicine, Polish Red Cross Maritime Hospital in Gdynia, Gdynia, POL; 5 Medicine, District Health Center Sp. z o.o. in Malbork, Malbork, POL; 6 Medicine, Florian Ceynowa Specialist Hospital, Wejherowo, POL; 7 Medicine, Nowy Szpital w Nakle i Szubinie, Szubin, POL; 8 Internal Medicine, Karol Marcinkowski University Hospital, Zielona Góra, POL

**Keywords:** gip receptor agonists, glp-1 receptor agonists, obesity, pharmacotherapy, semaglutide, tirzepatide, weight management

## Abstract

Obesity is a chronic and heterogeneous disease associated with a substantial burden of metabolic, cardiovascular, hepatic, and renal complications. In recent years, incretin-based therapies have changed the pharmacological management of obesity by providing levels of weight reduction that were previously difficult to achieve with medical treatment alone. This narrative review summarizes current evidence on the efficacy, safety, and broader clinical effects of glucagon-like peptide-1 (GLP-1) receptor agonists and dual glucose-dependent insulinotropic polypeptide (GIP)/GLP-1 receptor agonists in adults with overweight or obesity. Relevant literature was identified through searches of major medical databases, with emphasis on randomized controlled trials, meta-analyses, systematic reviews, clinical guidelines, and pivotal phase 2 and phase 3 trials. Liraglutide established the role of GLP-1 receptor agonism in weight management, while semaglutide provided greater and more sustained weight reduction. Tirzepatide, a dual GIP/GLP-1 receptor agonist, produced greater weight loss than semaglutide in direct comparative studies. Beyond weight reduction, incretin-based therapies have demonstrated favorable effects on glycemic control, cardiovascular risk, kidney outcomes, metabolic dysfunction-associated steatotic liver disease, and quality of life. Gastrointestinal adverse effects, particularly nausea, vomiting, and diarrhea, remain the most common limitations and are most prominent during dose escalation. Emerging multi-receptor and combination therapies, including retatrutide, CagriSema, MariTide, and amycretin, may further expand treatment options, although long-term efficacy and safety data remain limited. Overall, incretin-based therapies support a shift from weight-centered obesity treatment toward individualized, complication-oriented long-term care.

## Introduction and background

The global approach to weight management is undergoing a fundamental shift, moving away from the outdated view of obesity as a simple failure of willpower toward a recognition of its nature as a chronic, heterogeneous neurohormonal disease [1]. This review paper utilizes the Adiposity-Based Chronic Disease (ABCD) model to frame obesity as a neurohormonal-driven dysregulation of energy balance that results in abnormalities in adipose tissue mass and function (Figure 1). By focusing on this framework, the review transitions from a singular focus on body mass index to a complication-centric approach that prioritizes the prevention and mitigation of obesity-related conditions [1]. The following chapters trace the evolution of incretin-based therapies, from the early discovery of the "incretin effect" to the development of high-potency dual-receptor agonists, and evaluate their systemic impact on metabolic health [1,2]. This structural roadmap begins with the global epidemiological burden, explores the molecular mechanisms of GLP-1 and GIP activation, and concludes with a synthesis of clinical outcomes and future research priorities [2].

**Figure 1:**
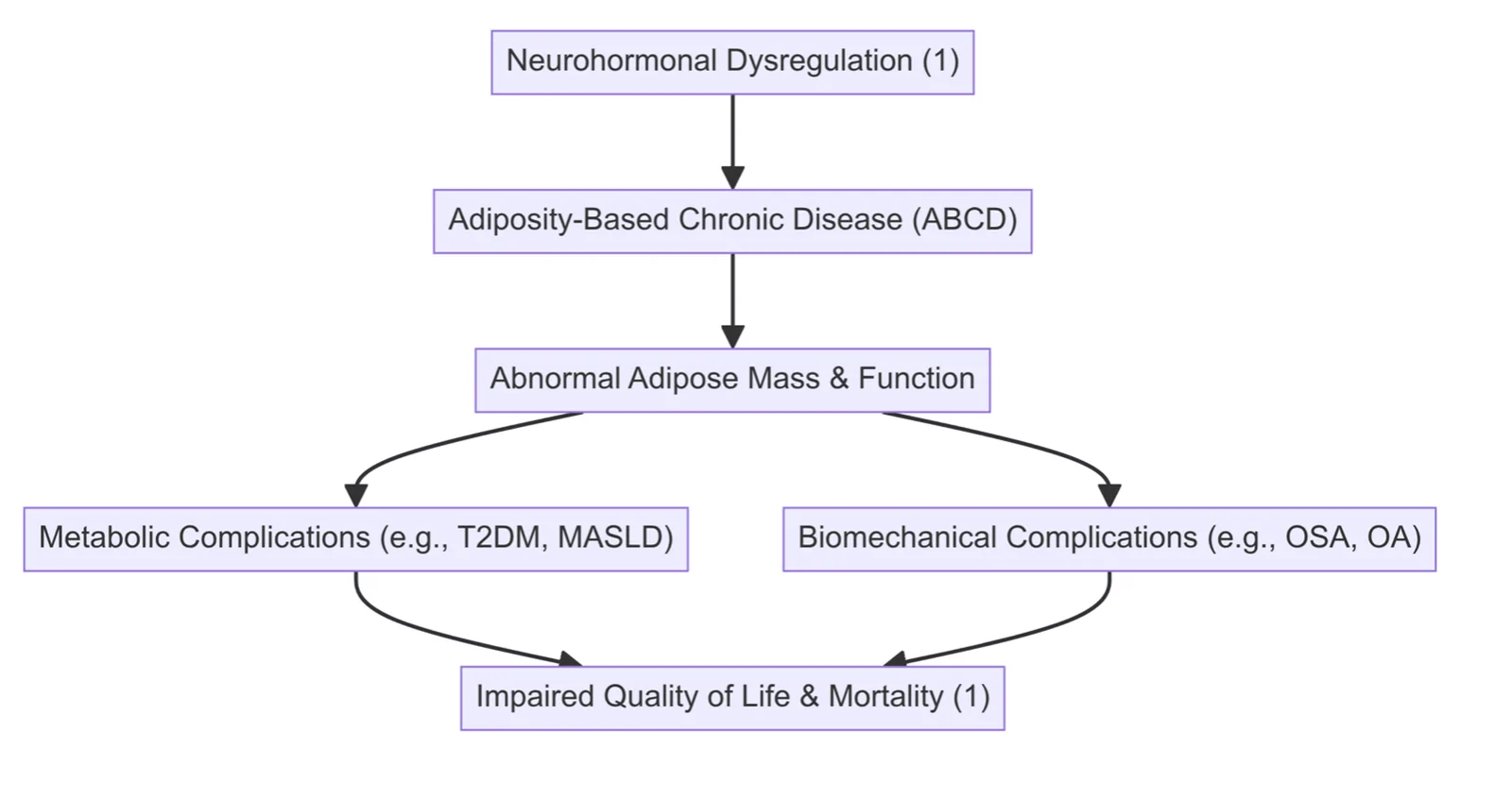
Conceptual Framework: Obesity as Adiposity-Based Chronic Disease (ABCD) Note: Neurohormonal dysregulation acts as the primary driver of energy imbalance. Adipose tissue mass and function are the phenotypic markers of the disease. MASLD: Metabolic dysfunction-associated steatotic liver disease, OSA: obstructive sleep apnea, T2DM: type 2 diabetes mellitus, OA: osteoarthritis. Source: Author’s own illustration based on [1].

Obesity as a global health problem

The global prevalence of obesity has reached unprecedented levels. It can no longer be treated as a lifestyle decision, but as a chronic neurohormonal disease. According to recent data from the World Health Organization, approximately 2.5 billion adults are overweight and about 890 million have obesity (Figure 2). Over the past four decades, the number of people with obesity has increased significantly. This trend is also evident across European Society of Cardiology member countries, where the prevalence of obesity is approximately 22.5% among adults. Although class 2 and class 3 obesity (BMI ≥35 kg/m²) have become increasingly prevalent, a substantial proportion of the population-level cardiovascular burden remains attributable to overweight and class 1 obesity [3]. This epidemic also places a substantial financial burden on healthcare systems [4]. The growing clinical and socioeconomic burden of obesity underscores the need for effective and sustainable therapeutic strategies. In adults, overweight is conventionally defined as a body mass index (BMI) of 25.0-29.9 kg/m² and obesity as a BMI of ≥30 kg/m². Although BMI remains widely used for screening and epidemiological classification, it does not directly measure adipose tissue distribution, function, or the presence of obesity-related complications. For this reason, contemporary frameworks increasingly complement BMI-based classification with assessment of clinical complications and adiposity-related health risk [1].

**Figure 2:**
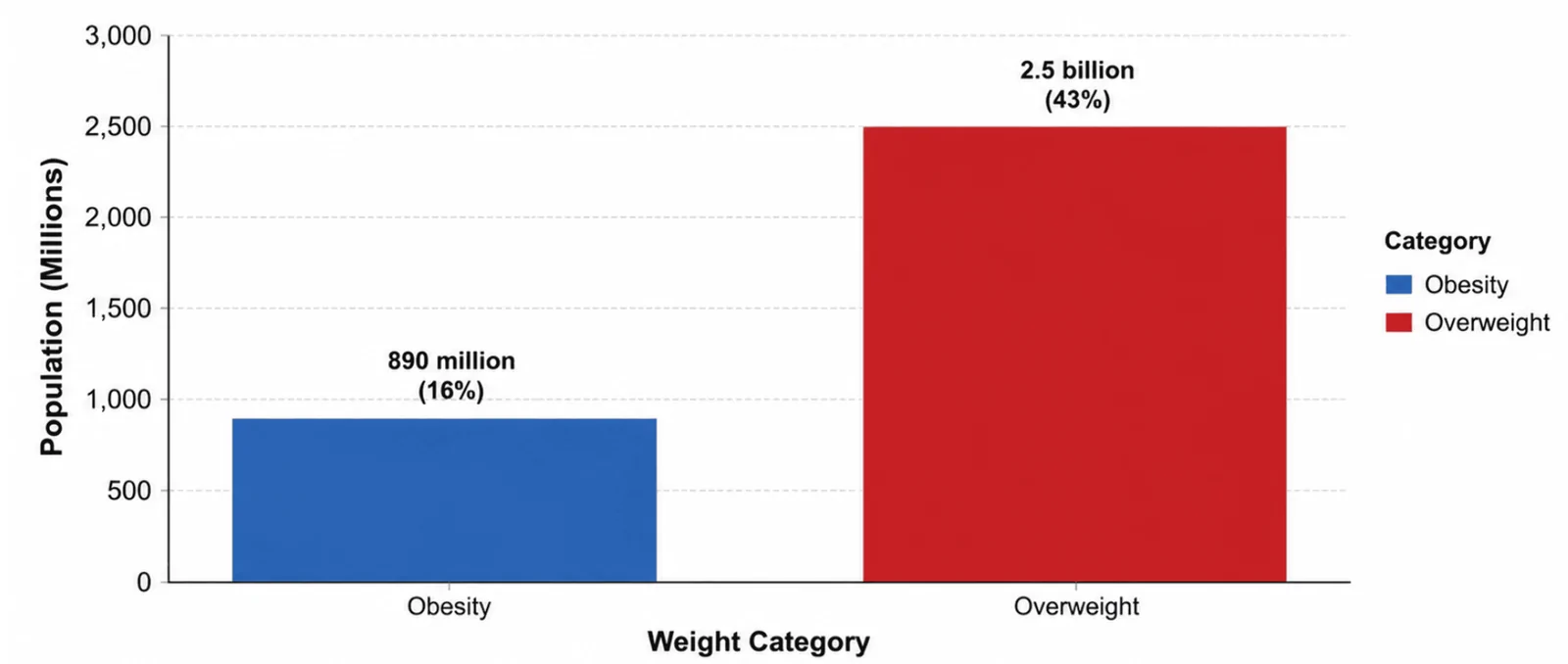
Global Obesity and Overweight Prevalence Note: Data represents adult populations aged 18 or older. Overweight includes individuals with BMI ≥ 25; Obese includes BMI ≥ 30. Source: Data from WHO estimates (2022).

Pathophysiology of obesity and the role of incretin hormones

The incretin effect is a basic biological mechanism in which oral nutrition initiates a stronger insulin response as compared to the same blood glucose response by intravenous administration [5]. The insulin response is mediated by the gut hormones, glucose-dependent insulinotropic polypeptide (GIP) and glucagon-like peptide-1 (GLP-1). They are released from K and L cells in the gastrointestinal tract after nutrients have been absorbed (Figure 3). Following nutrient ingestion, GIP and GLP-1 act as endocrine signals to the pancreatic islets and enhance glucose-dependent insulin secretion. GLP-1 also suppresses inappropriate postprandial glucagon secretion in a glucose-dependent manner and slows gastric emptying, thereby helping to attenuate postprandial glycemic excursions [5]. Together, these incretin-mediated effects contribute to the physiological regulation of postprandial glucose homeostasis. However, even in patients with chronic obesity, this effect is often substantially reduced, contributing to the metabolic dysfunction associated with the Adiposity-Based Chronic Disease framework [2,5].

**Figure 3:**
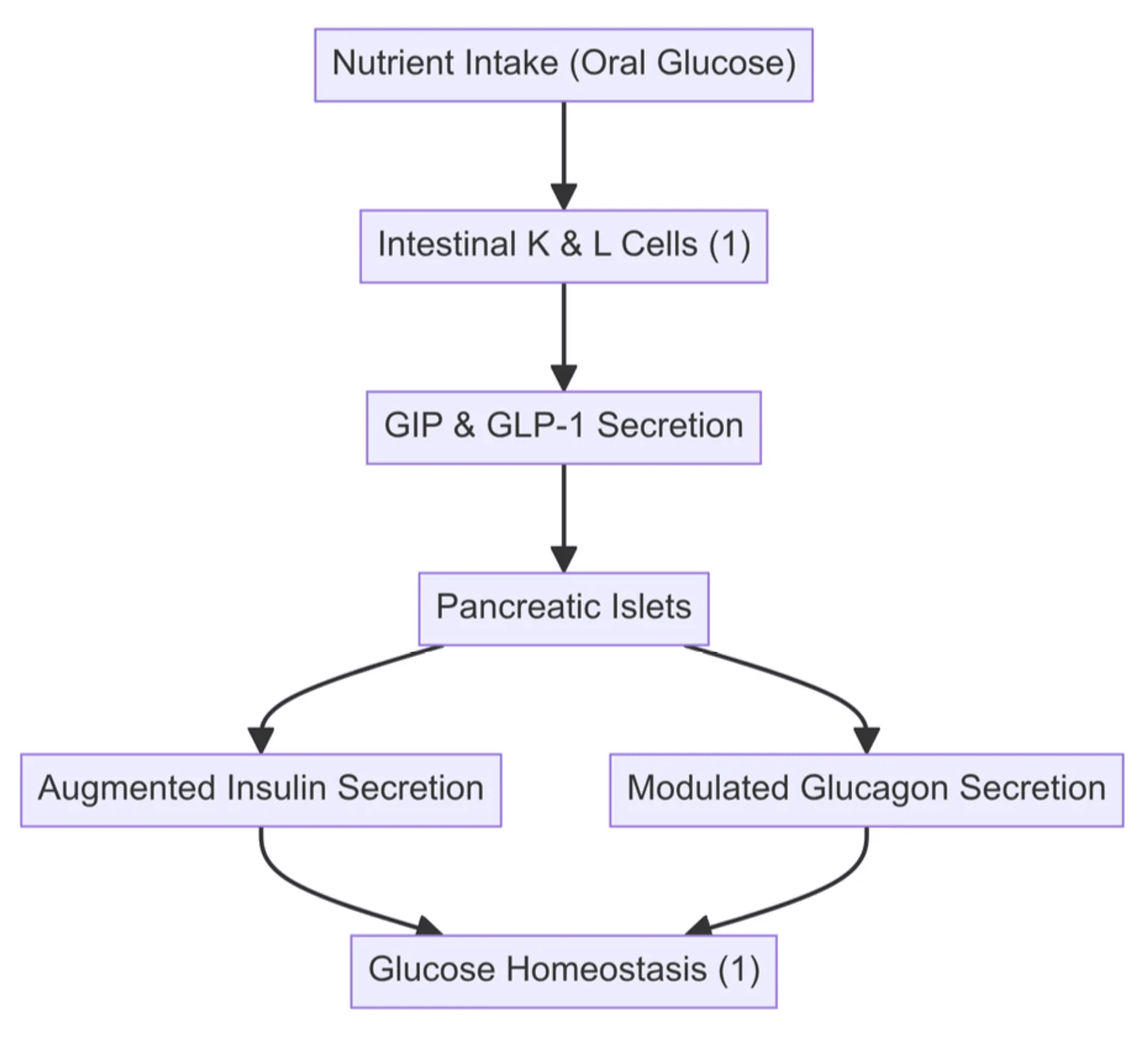
The Incretin Axis in Healthy Physiology Description: Schematic representation of the incretin effect, in which nutrient ingestion stimulates GIP and GLP-1 secretion and enhances glucose-dependent insulin secretion. Note: GLP-1 also suppresses inappropriate postprandial glucagon secretion and delays gastric emptying. GIP: glucose-dependent insulinotropic polypeptide, GLP-1: glucagon-like peptide-1. Source: Authors’ own illustration based on [2,5].

Current approaches to obesity treatment

Traditional weight management strategies typically integrate dietary modifications, increased physical activity, and psychological support to balance caloric intake and expenditure (Table 1). While these methods remain essential for primary prevention, they often yield only modest short-term weight reductions, typically ranging from 5% to 10% [3]. Clinical outcomes suggest that the long-term efficacy of these interventions is frequently undermined by the biological redundancy of food intake regulation, which necessitates targeting multiple signaling pathways for effective medical treatment [6]. Furthermore, evidence indicates that initial weight loss from most dietary patterns often diminishes after 12 months as patients struggle to maintain behavioral changes in an obesogenic environment [3]. This efficacy gap underscores the necessity for high-potency incretin mimetics, as traditional pharmacotherapies like orlistat offer limited weight reduction and lack robust cardiovascular safety data [3]. Consequently, while metabolic surgery remains the gold standard for achieving significant reductions of 25% to 30%, new pharmacological agents are increasingly positioned as viable alternatives to bridge this clinical gap [3,6]. In this context, the development of novel incretin-based therapies has substantially expanded pharmacological options for the treatment of obesity [7-10].

**Table 1:** Limitations of Traditional Obesity Management Note: Traditional drugs often lack robust cardiovascular safety data. Surgery remains the gold standard for high-potency weight loss. MACE: major adverse cardiovascular events, CV: cardiovascular. Source: Own aggregation based on [3].

Intervention	Typical Weight Loss	Primary Limitation	CV Impact
Lifestyle Modification	5%-10%	High rate of weight regain	Modest improvement
Traditional Drugs (e.g., Orlistat)	Modest	GI side effects/Low efficacy	Scarce safety data
Metabolic Surgery	25%-30%	Invasive/High-risk patients only	Significant protection
Incretin Mimetics (New)	15%-20%+	Cost/Long-term persistence	Proven MACE reduction

Aim of the review

Despite the rapid expansion of incretin-based pharmacotherapy, evidence regarding comparative efficacy, safety, systemic metabolic effects, and the clinical relevance of emerging multi-receptor agents remains distributed across different trials, populations, and therapeutic areas. The primary objective of this narrative review is therefore to synthesize current evidence on GLP-1 receptor agonists and dual GIP/GLP-1 receptor agonists in adults with overweight or obesity. Particular emphasis is placed on comparative weight-loss efficacy, treatment safety, clinically relevant obesity-related complications, and emerging therapeutic strategies. Where direct comparative evidence is available, differences between selective GLP-1 receptor agonism and dual GIP/GLP-1 receptor agonism are discussed, while cross-trial comparisons are interpreted cautiously in view of differences in study populations, treatment duration, and trial design.

## Review

Narrative review methodology

This narrative review was conducted to summarize current evidence regarding the efficacy, safety, metabolic benefits, and future perspectives of GLP-1 and dual GIP/GLP-1 receptor agonists in the treatment of obesity. A structured literature search was performed using PubMed, Embase, Web of Science, the Cochrane Library Database of Systematic Reviews, and Epistemonikos. The search focused on publications concerning GLP-1 receptor agonists, GIP/GLP-1 receptor agonists, obesity, weight reduction, metabolic outcomes, and treatment safety. Particular attention was given to randomized controlled trials, meta-analyses, systematic reviews, clinical guidelines, and major phase 2 and phase 3 clinical trials. Relevant recent publications were prioritized, while landmark studies were included when necessary to provide physiological or historical context. Only English-language publications relevant to the scope of this review were considered. The selected evidence was synthesized narratively according to the major clinical and pharmacological themes addressed in this review. No de novo meta-analysis, meta-regression, or independent statistical pooling was performed. Effect estimates, confidence intervals, and p-values reported in this review were derived from the original clinical trials, systematic reviews, and meta-analyses cited in the corresponding sections and were synthesized narratively.

Narrative literature search strategy

For this narrative review, a structured literature search was conducted across five medical databases: PubMed, Embase, Web of Science, the Cochrane Database of Systematic Reviews, and Epistemonikos (Table 2). This multi-database approach ensured a broad coverage of high-quality clinical outcomes, particularly those derived from multi-center trials and systematic meta-analyses. To ensure the inclusion of the most current therapeutic advancements, temporal boundaries were established to prioritize recent data, with search activities extending through August 2026 to capture the latest phase 3 trial results. Specific search phrases utilized included MeSH descriptors such as “Tirzepatide” and “GLP-1 RAs,” combined with terms for “systematic review” and “meta-analysis” to isolate the highest levels of clinical evidence. Furthermore, language filters were applied to focus exclusively on English-language publications, which helped maintain methodological consistency and ensured the analysis was based on international standards of clinical reporting. This search strategy was designed to identify relevant high-quality evidence and support a structured narrative synthesis of the efficacy and safety of modern incretin-based therapies.

**Table 2:** Literature Search Strategy Description: Summary of the structured literature search strategy used to identify evidence regarding GLP-1 and GIP/GLP-1 receptor agonists in obesity treatment. Note: Priority was given to high-quality clinical evidence, including randomized controlled trials, systematic reviews, meta-analyses, clinical guidelines, and pivotal phase 2 and phase 3 trials. GLP-1: glucagon-like peptide-1, GIP: glucose-dependent insulinotropic polypeptide, MASLD: metabolic dysfunction-associated steatotic liver disease. Source: Authors’ own elaboration.

Component	Description
Databases searched	PubMed, Embase, Web of Science, Cochrane Library, and Epistemonikos
Main search concepts	Obesity, overweight, incretin-based therapy, GLP-1 receptor agonists, GIP/GLP-1 receptor agonists
Drug-related terms	Liraglutide, semaglutide, tirzepatide, retatrutide, CagriSema, MariTide, amycretin
Efficacy-related terms	Weight loss, body weight reduction, obesity treatment, metabolic outcomes, glycemic control
Safety-related terms	Safety, adverse events, gastrointestinal adverse effects, tolerability, treatment discontinuation
Additional clinical outcomes	Cardiovascular outcomes, type 2 diabetes, MASLD, chronic kidney disease, quality of life
Preferred evidence	Randomized controlled trials, systematic reviews, meta-analyses, clinical practice guidelines, and large observational studies
Population of interest	Adults with overweight or obesity, including individuals with obesity-related metabolic complications
Language	English
Additional identification of studies	Reference lists of relevant reviews, clinical guidelines, and pivotal clinical trials were screened for additional eligible publications
Evidence synthesis	Relevant evidence was synthesized narratively according to pharmacological and clinical themes

Eligibility criteria and study selection

Publications were eligible for inclusion if they addressed the pharmacological treatment of overweight or obesity with GLP-1 receptor agonists or dual GIP/GLP-1 receptor agonists. Randomized controlled trials, systematic reviews, meta-analyses, clinical practice guidelines, and large observational studies were included, as well as studies that evaluated efficacy, safety, cardiovascular, metabolic, renal, hepatic, and quality-of-life outcomes. Studies based on preclinical models or unrelated purposes were not considered except in the cases where mechanistic evidence was needed to explain the pharmacological effects of incretin-based therapies. Study selection was performed for the purposes of a narrative synthesis rather than a formal systematic review. No Preferred Reporting Items for Systematic Reviews and Meta-Analyses (PRISMA)-based screening procedure or standardized risk-of-bias assessment was undertaken. Studies were selected according to their relevance to the prespecified clinical and pharmacological themes of the review, with priority given to randomized controlled trials, systematic reviews, meta-analyses, clinical guidelines, and pivotal phase 2 and phase 3 studies.

GLP-1 and GIP receptor agonists in obesity treatment

Modern obesity management has entered a new era defined by precision neurohormonal targeting, moving away from the limited efficacy of earlier lifestyle and pharmacological models. This chapter examines the transition from established long-acting selective GLP-1 receptor agonists to the innovative class of unimolecular dual agonists. The following sections provide a structured analysis starting with the molecular mechanisms of action, followed by a review of mono-agonist benchmarks like liraglutide and semaglutide. The discussion then focuses on the clinical breakthrough of dual-receptor innovation, specifically tirzepatide, before concluding with a synthesis of comparative metabolic outcomes and efficacy hierarchies.

Mechanism of action

The molecular foundation of modern obesity therapy rests on the activation of two distinct yet structurally related class B G protein-coupled receptors: the GLP-1 and GIP receptors. These receptors share a common architecture within the glucagon receptor superfamily, characterized by a specific binding mechanism where the hormone’s C-terminal interacts with an extracellular domain, while the N-terminal engages the transmembrane region [11,12]. Once activated, both receptors utilize a canonical signaling pathway that triggers adenylyl cyclase to produce cyclic adenosine monophosphate (cAMP), subsequently activating regulators such as exchange protein directly activated by cyclic adenosine monophosphate (EPAC) and protein kinase A (PKA) [13]. Tirzepatide has been reported to exhibit biased signaling at the GLP-1 receptor, favoring cAMP signaling relative to beta-arrestin recruitment [2]. This signaling profile has been proposed as a potential mechanism that may influence receptor internalization, recycling, and downstream insulinotropic effects [13]. However, the extent to which biased agonism directly explains the superior clinical efficacy observed with tirzepatide remains uncertain, and these mechanistic observations should not be interpreted as an established explanation for clinical outcomes.

The regulation of body weight by incretin mimetics is primarily driven by their action within the central nervous system. GLP-1 receptor agonists target multiple brain regions, including the hypothalamus and the area postrema, where they activate satiety-stimulating neurons to reset body weight setpoints [12]. This neuroanatomical distribution is extensive, with Hammoud [11] noting that neurons in at least 10 different regions transduce these signals to reduce food intake. The synergy of dual-receptor activation is illustrated in Figure 4, which shows how GLP-1 and GIP target non-redundant and complementary pathways. Experimental studies suggest that GIP receptor signaling may modulate anorexigenic pro-opiomelanocortin (POMC) neuronal pathways and thereby contribute to the regulation of food intake [12]. Preclinical evidence also suggests that GIP receptor activation may influence neuronal pathways within the area postrema that are involved in nausea associated with GLP-1 receptor activation [11,13]. However, the clinical relevance of these mechanisms in humans remains incompletely established. These findings provide a potential neurobiological explanation for some of the effects observed with dual GIP/GLP-1 receptor agonism, but they should be interpreted as mechanistic hypotheses rather than confirmed clinical mechanisms.

**Figure 4:**
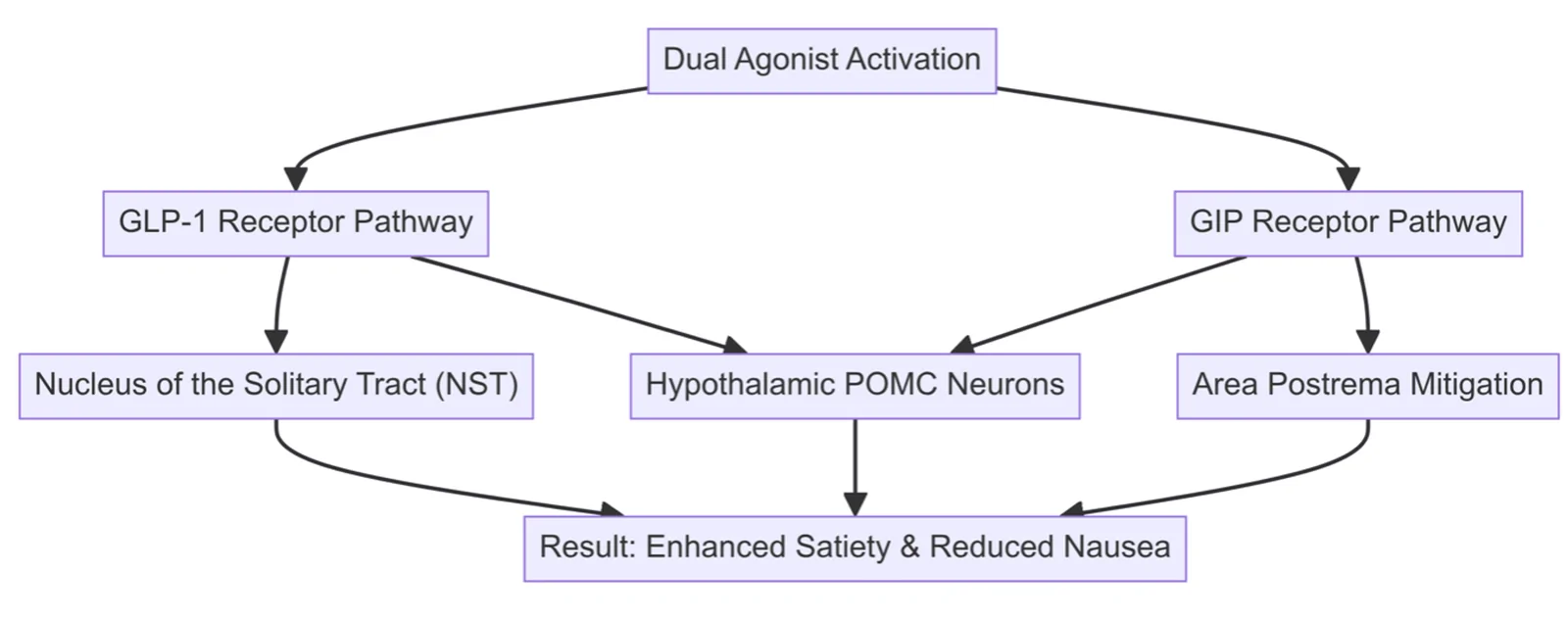
Molecular Synergy of GLP-1 and GIP Receptor Activation Description: Conceptual model of the non-redundant and complementary pathways activated by dual incretin receptor agonism. Note: GLP-1 signaling involves central pathways regulating satiety. Experimental evidence suggests that GIP receptor signaling may modulate POMC-related pathways and neuronal circuits involved in nausea; the clinical relevance of these mechanisms in humans remains under investigation. GIP: glucose-dependent insulinotropic polypeptide, GLP-1: glucagon-like peptide-1, POMC: pro-opiomelanocortin. Source: Author’s own illustration based on [11-13].

Beyond central satiety, incretins exert significant peripheral effects that contribute to metabolic homeostasis. GLP-1 acts as a potent inhibitor of gastric emptying, which helps reduce postprandial glucose surges, although Drucker and Holst [2] note that this effect is subject to rapid tachyphylaxis. In contrast, GIP plays a unique and non-redundant role in adipose tissue by enhancing lipoprotein lipase activity and improving healthy lipid storage [12]. These tissue-specific actions are detailed in Table 3, which compares the physiological roles of both receptors across various organ systems. Dual agonism may also influence systemic signaling pathways, including phosphoinositide 3-kinase/protein kinase B (PI3K/Akt) and extracellular signal-regulated kinase (ERK) cascades, that have been implicated in anti-inflammatory and organ-protective effects [13]. However, the extent to which these mechanisms contribute to clinical organ protection independently of weight loss remains uncertain. By combining central appetite suppression with direct peripheral effects on insulin sensitivity and lipid buffering, dual-receptor agonists like tirzepatide represent a transition toward holistic, multi-organ metabolic stabilization [11]. This comprehensive pharmacological profile may contribute to therapeutic benefits beyond BMI reduction to broader systemic health improvements.

**Table 3:** Tissue-Specific Effects of GIP and GLP-1 Description: Distribution and physiological roles of incretin receptors in metabolic organs. Note: GLP-1 is a potent inhibitor of gastric emptying (peripheral). GIP plays a unique role in adipose tissue lipid buffering. GIP: glucose-dependent insulinotropic polypeptide, GLP-1: glucagon-like peptide-1, MACE: major adverse cardiovascular events, LPL: lipoprotein lipase. Source: Based on [2,11-13].

Organ System	GLP-1 Receptor Action	GIP Receptor Action
Brain (CNS)	Satiety, reduced food reward	Energy expenditure; potential modulation of nausea-related pathways
Pancreas	Glucose-dependent insulin secretion	≈50% of total incretin effect
Stomach	Delayed gastric emptying	No significant effect
Adipose Tissue	Reduced inflammation	Enhanced lipid buffering and LPL activity
Heart	Cardioprotection, MACE reduction	Expression in ventricular myocardium

GLP-1 receptor agonists (liraglutide, semaglutide)

The transition of liraglutide from a glycemic control agent for diabetes to a primary treatment for obesity marked a significant regulatory milestone in 2014 [14]. As a long-acting analog with 87% homology to human glucagon-like peptide 1, liraglutide functions primarily by suppressing appetite and delaying gastric emptying [14]. Meta-analytical data from 31 randomized controlled trials indicate that this pharmacological intervention consistently achieves a mean weight reduction of 4.19 kg, representing an average change of 4.16% from baseline [14]. Due to its 13-hour half-life, maintaining these physiological effects requires a once-daily subcutaneous administration [14]. While these outcomes are modest compared to newer agents, liraglutide established the foundational evidence for the safety and effectiveness of selective receptor agonism in weight management across both diabetic and non-diabetic populations.

Semaglutide is an analog of long-acting GLP-1, mainly used to control T2DM, and is also suitable for promoting weight loss in people with mild obesity [15]. The development of once-weekly semaglutide 2.4 mg represents a substantial advancement in the potency of mono-agonist therapy. Data from the STEP 5 trial demonstrate that participants achieved a mean body weight change of -15.2% over a 104-week period, significantly outperforming the -2.6% reduction observed in the placebo group [16]. This high level of efficacy is further reflected in categorical success, with approximately 36.1% of participants reaching a high-magnitude weight loss threshold of 20% or more [15,16]. Unlike many traditional interventions that suffer from early weight regain, semaglutide-induced weight loss typically reaches a plateau around week 60 and remains sustained through 104 weeks when combined with adjunct behavioral support [16]. Once-weekly semaglutide 2.4 mg provides greater weight reduction than earlier selective GLP-1 receptor agonists while offering the convenience of weekly administration.

The shift from daily to weekly dosing was made possible by structural modifications, such as palmitoyl chains or polyethylene glycol (PEG) conjugation, which allow these molecules to resist dipeptidyl peptidase-4 (DPP-4) degradation and extend their therapeutic window [15]. Beyond simple weight reduction, high-potency selective agonists provide comprehensive cardiometabolic improvements, including significant decreases in waist circumference and systolic blood pressure [14,16]. For instance, semaglutide 2.4 mg is associated with a systolic blood pressure reduction of 5.7 mmHg [16]. Furthermore, these therapies support metabolic reversal in high-risk groups; nearly 80% of participants with prediabetes at baseline reverted to normoglycemia during long-term treatment [16]. By targeting both central satiety and peripheral metabolic pathways, these selective agonists establish a robust clinical baseline, although their use is often balanced against dose-dependent gastrointestinal side effects observed during the initial titration phase [15].

Dual GLP-1/GIP receptor agonists (tirzepatide)

Tirzepatide is the first dual GIP/GLP-1 receptor agonist and has demonstrated beneficial effects on glycemic control, body weight, and cardiovascular risk factors [8]. Tirzepatide is defined in clinical literature as a once-weekly injectable peptide that simultaneously activates both glucose-dependent insulinotropic polypeptide (GIP) and glucagon-like peptide-1 (GLP-1) receptors [17]. Structurally, the molecule is a 39-amino-acid linear peptide derived from the human GIP sequence, representing a technological shift from early multi-receptor peptides toward unimolecular co-agonism [6]. This dual targeting approach is hypothesized to be more effective than targeting a single receptor because the patterns of receptor expression in the brain do not fully overlap [7]. Furthermore, evidence suggests that GIP receptors located on adipocytes may allow for the direct regulation of fat tissue by tirzepatide, a mechanism absent in selective GLP-1 mono-agonists [7]. By engaging both pathways within a single molecule, tirzepatide aims to replicate natural gut signaling more effectively than previous generations of metabolic pharmacotherapy.

The clinical efficacy of tirzepatide has been evaluated across the SURMOUNT trial program in populations with differing metabolic characteristics. In SURMOUNT-2, which enrolled adults with obesity or overweight and type 2 diabetes, tirzepatide 10 mg and 15 mg produced mean body weight reductions of 12.8% and 14.7%, respectively [17]. In contrast, SURMOUNT-5 provided direct head-to-head evidence comparing tirzepatide with semaglutide in a different study population and reported mean weight changes of -20.2% and -13.7%, respectively [7]. Because weight-loss responses may differ according to diabetes status, trial duration, dose, and baseline characteristics, results from separate trials should not be interpreted as directly interchangeable. Categorical success is also notable, as 31% of participants on the 15 mg dose in diabetic cohorts achieved a weight reduction of 20% or more [17]. As illustrated in Figure 5, the high percentage of patients reaching these significant thresholds underscores the drug’s potency. These results suggest that dual GIP/GLP-1 agonism allows for weight-loss levels that approach those traditionally only achievable through metabolic surgery.

**Figure 5:**
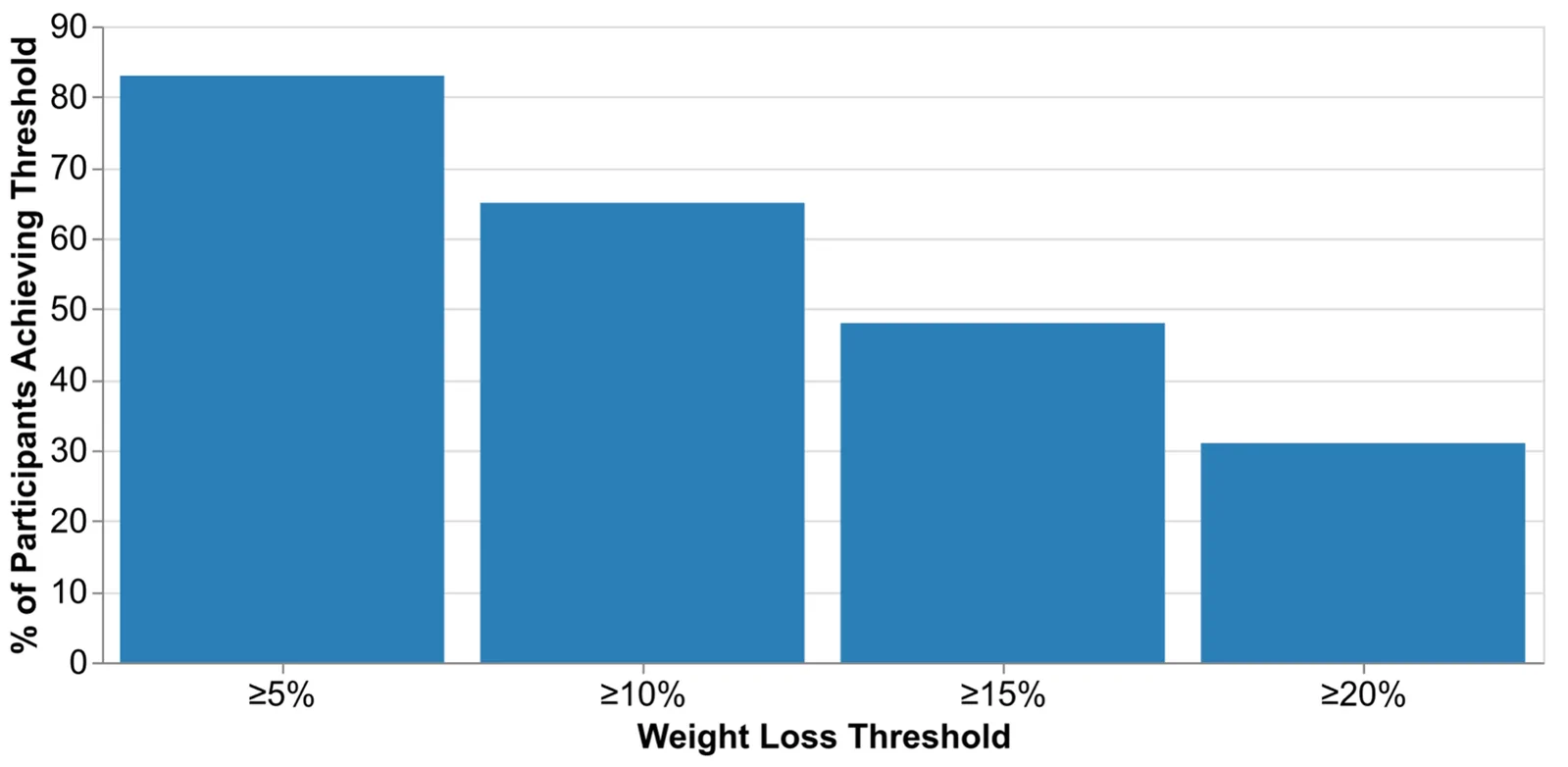
Categorical Weight Loss Success With Tirzepatide Description: Percentage of patients achieving specific weight loss thresholds with tirzepatide 15 mg in the SURMOUNT-2 trial. Note: Data represents the 15 mg dose group in patients with type 2 diabetes. Nearly one-third of patients achieved the high-tier threshold of ≥20% weight loss. Source: Data from [17].

Beyond mass reduction, tirzepatide provides substantial metabolic improvements, particularly in glycemic control. Treatment in diabetic populations led to an average HbA1c reduction of approximately 2%, with nearly half of participants reaching normoglycemia below 5.7% [17]. The drug also demonstrates a superior metabolic impact compared to mono-agonists, including a systolic blood pressure reduction of 10.2 mmHg versus 7.7 mmHg for semaglutide [7]. Physiological studies using hyperinsulinemic-euglycemic clamps indicate that tirzepatide enhances insulin sensitivity to a greater degree than selective agents, an effect that appears to be partially independent of the absolute magnitude of weight lost [6]. This suggests that unique GIP receptor signaling effects contribute directly to alpha-cell regulation and systemic glucose homeostasis [6]. These findings support the use of dual agonists as a comprehensive tool for managing both adiposity and its related metabolic complications.

Comparative efficacy and effects on metabolic outcomes

Establishing a clear hierarchy of efficacy is essential for clinical decision-making in obesity management. Direct head-to-head evidence from the SURMOUNT-5 trial indicates that tirzepatide achieves a significantly greater mean weight reduction of -20.2% compared to -13.7% for semaglutide over 72 weeks [7]. In the cited 40-week comparison, mean body weight reduction was -12.4 kg with tirzepatide and -6.2 kg with semaglutide [13]. These findings should be interpreted in the context of the specific study population, doses, and follow-up period rather than as evidence that tirzepatide is universally twice as effective as semaglutide. Furthermore, in the SURMOUNT-5 trial, participants receiving tirzepatide were approximately twice as likely to achieve a weight reduction of 25% or more compared with those receiving semaglutide [7]. These head-to-head findings demonstrate greater weight reduction with tirzepatide than with semaglutide under the specific conditions of this trial, although the magnitude of the difference should be interpreted in the context of the study population, doses, and duration of treatment.

The comparative metabolic impact of these agents is particularly evident in the restoration of glycemic control. Dual GIP/GLP-1 co-agonism reduces HbA1c and body weight in individuals with type 2 diabetes more effectively than selective GLP-1 agonists like semaglutide [18]. This physiological advantage likely stems from the fact that while GIP mono-therapy often shows no significant effect in previously hyperglycemic patients, its combination with GLP-1 appears necessary for significant glucose lowering [18]. Modern pharmacotherapy thus enables a treat-to-target approach, where achieving normoglycemia and high-magnitude weight loss of 15% to 20% becomes a feasible clinical goal [7]. By reaching these thresholds, clinicians can more effectively target the remission of obesity-related comorbidities, shifting the focus from simple BMI management to comprehensive glycemic restoration.

Beyond mass reduction, these therapies offer significant vascular protection through the improvement of cardiometabolic risk markers. Tirzepatide demonstrates superior improvements in systolic blood pressure compared to semaglutide, as illustrated in Figure 6. Specifically, high doses of tirzepatide can reduce systolic blood pressure by as much as 12.6 mmHg [7,13]. These metabolic benefits are dose-dependent and correlate with the magnitude of weight loss, leading to significant reductions in atherogenic lipid particles such as low-density lipoprotein particles (LDLP) and triglyceride-rich lipoprotein particles (TRLP) [7,13]. Furthermore, potent systemic anti-inflammatory effects, evidenced by reductions in biomarkers like C-reactive protein (CRP) and intercellular adhesion molecule-1 (ICAM-1), likely contribute to improved endothelial function and a lower risk of atherosclerotic progression [13]. These findings suggest that the clinical benefits of dual agonists may extend beyond body weight reduction and include broader cardiometabolic effects.

**Figure 6:**
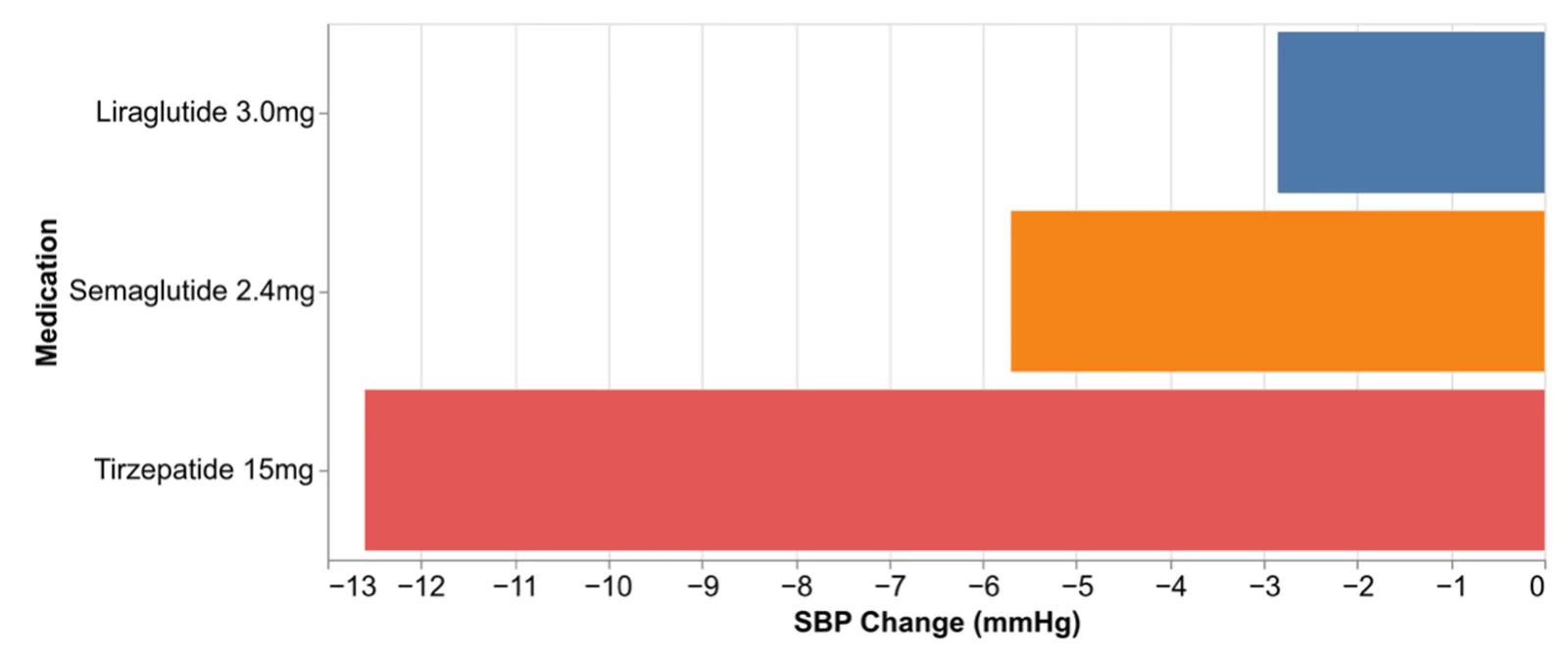
Blood Pressure Reduction: Dual vs. Mono-Agonists Description: Comparative reduction in systolic blood pressure (SBP) across major clinical programs. Note: Tirzepatide 15 mg showed significantly greater SBP reduction compared to traditional GLP-1RAs. These improvements may reflect both weight-loss-related and additional cardiometabolic effects. GLP-1RAs: glucagon-like peptide-1 receptor agonists. Source: Data from [13,16].

Understanding the temporal dynamics of weight loss is critical for setting realistic patient expectations. Long-term data from the SELECT trial indicates that semaglutide-induced weight loss typically continues until week 65 before reaching a sustained plateau that can be maintained for up to four years [19]. This settling point represents a body weight in harmony with genetic and environmental determinants, marking the physiological limit of the therapy’s effect [19]. Baseline BMI significantly influences these plateaus; individuals with lower baseline obesity may experience smaller percentage reductions while still achieving vital visceral fat loss [19]. These findings emphasize that weight loss cannot continue indefinitely, and the achievement of a stable plateau should be viewed as a successful physiological adaptation rather than a failure of the pharmacological intervention.

Several molecular mechanisms have been proposed to contribute to the clinical effects of dual agonists. Biased agonism at the GLP-1 receptor, including altered receptor internalization and recycling, has been suggested as one potential mechanism that may influence the durability of metabolic signaling [18]. However, a direct causal relationship between this signaling profile and the superior clinical efficacy of tirzepatide has not been established. Improved insulin sensitivity during dual GIP/GLP-1 receptor agonist therapy may not be fully explained by weight loss alone, and direct GIP-mediated effects in adipose tissue and the pancreas have been proposed as potential contributing mechanisms [13]. Clinically, tirzepatide has also been associated with greater reductions in abdominal adiposity; for example, an additional 5.4 cm reduction in waist circumference compared with semaglutide was reported in the cited trial [7]. These findings suggest that multiple central and peripheral pathways may contribute to the metabolic effects of dual-receptor agonism, although their relative contribution to the observed clinical efficacy remains uncertain.

Safety and clinical benefits

Modern obesity management is undergoing a significant transition as the focus moves from simple body mass index reduction toward a holistic model of organ protection and metabolic health. This chapter frames the essential benefit-risk clinical assessment, balancing the frequent challenges of gastrointestinal tolerability against the potential for substantial reductions in long-term mortality. As illustrated in Figure 7, this conceptual framework establishes that the ultimate goal of therapy is achieving metabolic health within the Adiposity-Based Chronic Disease (ABCD) model. The following sections provide a structured orientation on the safety profiles and common adverse effects, followed by a detailed analysis of the systemic benefits across different organ systems.

**Figure 7:**
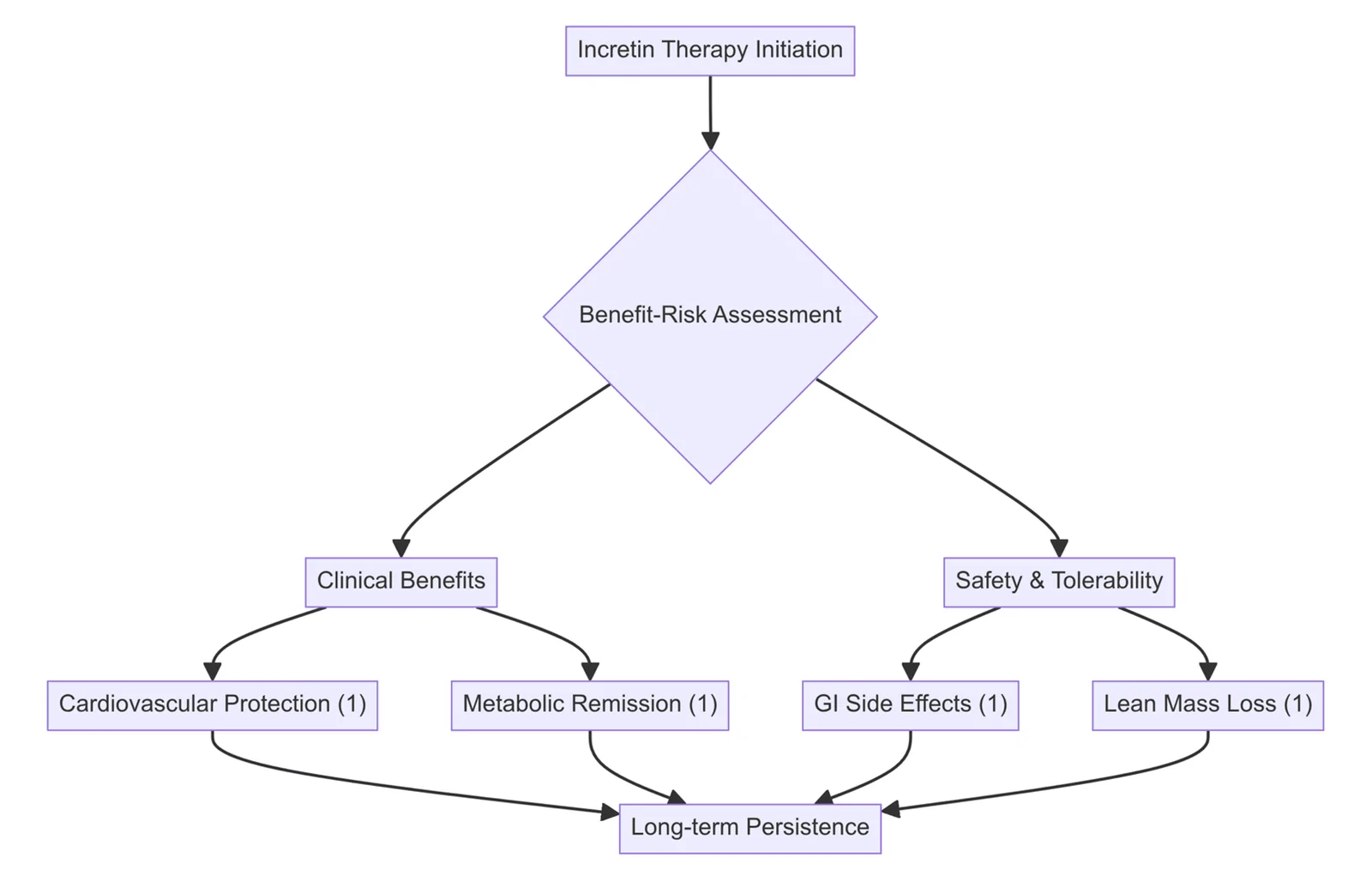
Benefit-Risk Framework for Incretin-Based Obesity Therapy Description: Conceptual framework illustrating the balance between metabolic benefits and treatment-related tolerability in incretin-based obesity therapy. Note: The primary goal is organ protection rather than just BMI reduction. Gastrointestinal side effects are the most frequent barrier to long-term persistence. Source: Author’s own illustration based on [1,20].

Safety profile and adverse effects

Incretin-based therapies, including both selective GLP-1 receptor agonists and dual GIP/GLP-1 agonists like tirzepatide, are associated with a distinct class effect of gastrointestinal symptoms. Systematic reviews indicate that nausea, vomiting, and diarrhea represent the most frequent side effects [8,9,20,21]. These events are predominantly dose-dependent and peak during the initial titration phase, which is a near-universal barrier across clinical trials [1]. Other side effects reported by patients include intestinal obstruction and pancreatitis [9]. As detailed in Table 4, these common adverse events are the primary drivers of treatment discontinuation. To mitigate clinically significant distress, clinical guidelines emphasize a conservative initiation at the lowest possible dose followed by slow monthly up-titration [1]. This structured escalation, illustrated in Figure 8, is vital for allowing the body to acclimate to the neurohormonal changes, thereby improving long-term patient persistence. Careful titration remains the cornerstone of managing the ubiquitous gastrointestinal distress that characterizes the early stages of high-potency metabolic pharmacotherapy [1].

**Table 4:** Common and Rare Adverse Events of GLP-1 and GIP/GLP-1 Agonists Description: Comparison of frequent gastrointestinal side effects and rare safety concerns based on clinical trial data. Note: GI symptoms are usually transient and peak during dose escalation. Randomized clinical trials have not demonstrated a clear increased risk of acute pancreatitis; however, pancreatitis remains an important safety consideration in clinical practice. GIP: glucose-dependent insulinotropic polypeptide, GLP-1: glucagon-like peptide-1, BMD: bone mineral density, MEN2: multiple endocrine neoplasia type 2. Source: Data from [1,8,9,20,21].

Adverse Event Type	Specific Symptoms/Conditions	Frequency/Risk Level	Clinical Implication
Gastrointestinal	Nausea, vomiting, diarrhea	15%-45% (Very Common)	Primary cause of discontinuation
Skeletal/Muscle	Loss of lean mass, BMD reduction	~25%-39% of weight loss	Risk of sarcopenia in elderly
Biliary	Gallstones, cholecystitis	Slightly elevated	Monitor during rapid weight loss
Pancreatic	Acute pancreatitis	Inconclusive/Rare	No causal link in large trials
Thyroid	Medullary thyroid carcinoma	Rare (Rodent-specific)	Contraindicated in MEN2/Family history

**Figure 8:**
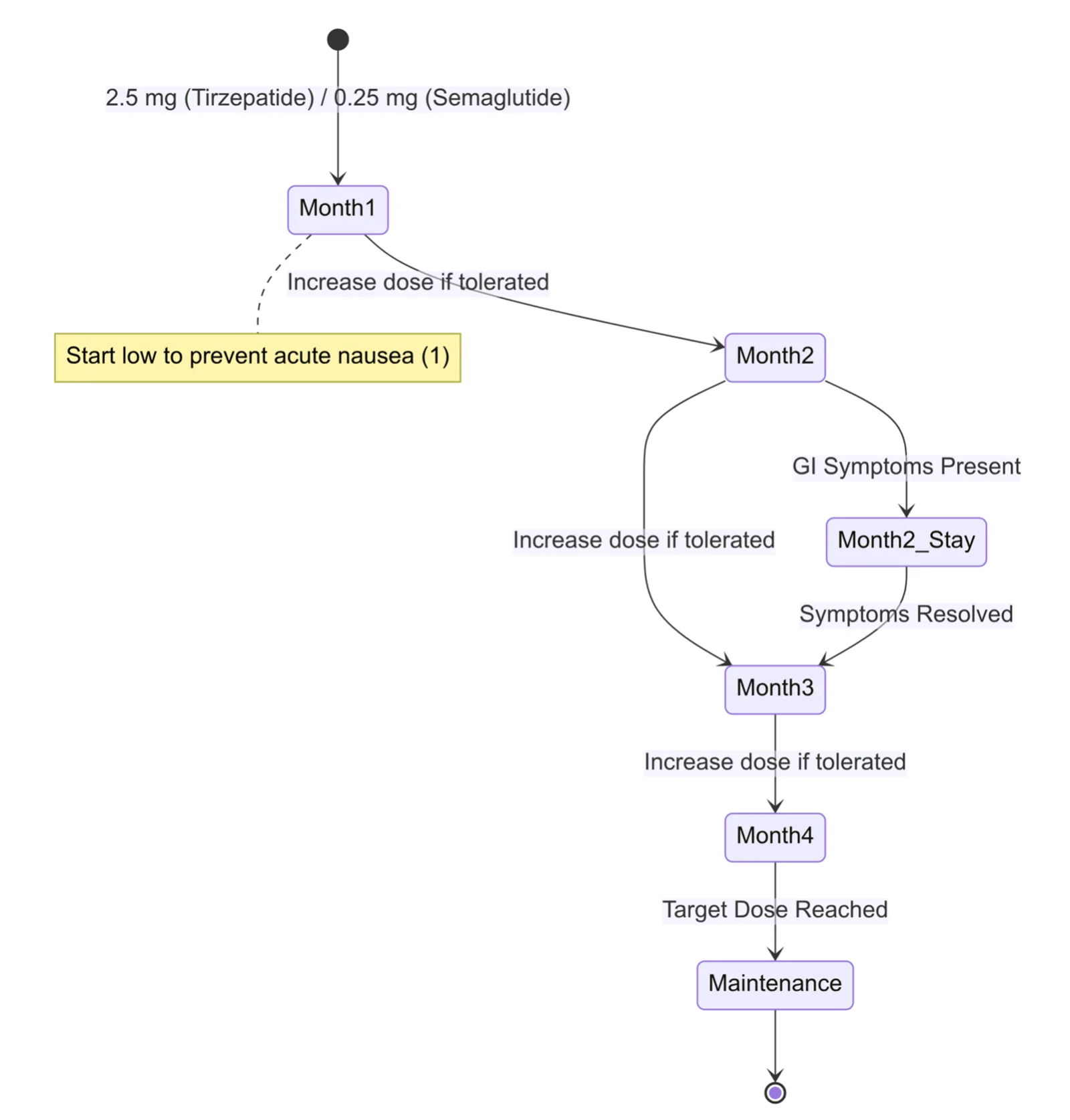
Standard Dose Titration Protocol for GLP-1/GIP Agonists Description: Clinical escalation schedule designed to mitigate gastrointestinal side effects and improve patient adherence. Note: Dose increments typically occur every four weeks. Slower titration is recommended if GI symptoms persist. GIP: glucose-dependent insulinotropic polypeptide, GLP-1: glucagon-like peptide-1. Source: Author’s own illustration based on [1].

While common side effects are transient, clinicians must remain vigilant for rare but serious safety signals. Early rodent data raised concerns regarding medullary thyroid carcinoma, yet human relevance appears limited because human C-cells express significantly fewer GLP-1 receptors [20]. Nevertheless, a history of multiple endocrine neoplasia type 2 (MEN2) remains a strict contraindication. Specialized meta-analyses suggest a slightly elevated risk of gallbladder and biliary diseases with prolonged treatment, although the link to acute pancreatitis remains inconclusive in long-term cardiovascular trials [1,21]. Furthermore, GLP-1 receptor agonists cause a modest increase in heart rate. As summarized in Table 4, specialized monitoring is required for patients experiencing severe vomiting or diarrhea to prevent dehydration-related renal impairment [1]. Additionally, GLP-1 receptor agonists significantly delay gastric motility, with one study showing a 56% incidence of retained gastric contents, which necessitates careful perioperative planning to avoid aspiration pneumonia during anesthesia [20]. These rare risks require a personalized benefit-risk assessment, particularly in patients with pre-existing organ dysfunction.

A significant clinical challenge of rapid weight loss with high-potency incretins is the accompanying reduction in fat-free mass. Data indicate that lean mass loss accounts for approximately 39% of total weight reduction with semaglutide and 25% with tirzepatide [20]. This trend, visualized in Figure 9, raises concerns regarding sarcopenia and long-term physical functioning, especially in older adults. Furthermore, liraglutide has been associated with a 1% loss of bone mineral density in the femur and spine after one year, highlighting the need for skeletal health monitoring [21]. These physiological changes occur alongside substantial challenges in treatment persistence. Under real-world conditions, two-year persistence with GLP-1 receptor agonists is notably low at 48.4%, significantly trailing the 72.7% observed for other agents [22]. This disparity between clinical trials and real-world practice is driven by age, cost, and gastrointestinal distress [20]. Consequently, incorporating resistance training and monitoring body composition is essential to preserve functional health during chronic therapy [1].

**Figure 9:**
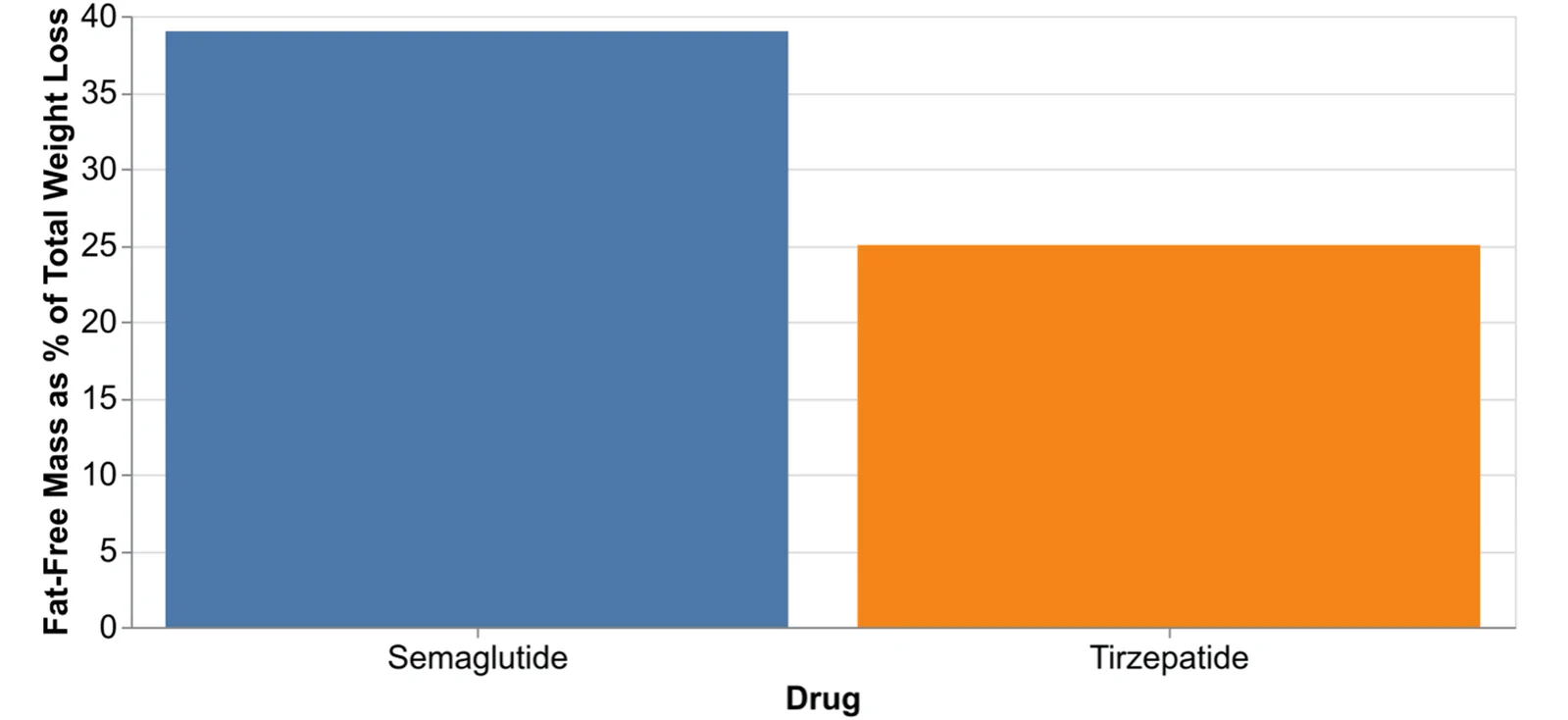
Body Composition: Fat-Free Mass Loss Description: Proportion of total weight loss derived from fat-free (lean) mass for different incretin-based therapies. Note: Loss of lean mass is a significant concern for elderly patients (sarcopenia risk). Tirzepatide showed a lower proportion of lean mass loss compared to semaglutide in this study. Source: Data from [20].

Benefits beyond weight loss

Beyond their effects on body weight, incretin-based therapies have been associated with clinically relevant metabolic and organ-specific outcomes. The following sections summarize evidence relating to type 2 diabetes, cardiovascular disease, metabolic dysfunction-associated steatotic liver disease, chronic kidney disease, and quality of life.

Type 2 diabetes mellitus

Modern incretin-based pharmacotherapy has demonstrated clinically relevant effects on the prevention of type 2 diabetes, particularly in individuals achieving substantial weight reduction. Long-term results from the SURMOUNT-1 trial indicate that tirzepatide reduced the risk of progression from prediabetes to type 2 diabetes by 93% over a 176-week period [10]. This high level of efficacy is mirrored by selective agonists, which enable a large majority of prediabetic patients to revert to normoglycemia. For instance, data suggest that 81% of participants reached healthy HbA1c levels with semaglutide compared to only 14% with placebo [10]. Even first-generation agents like liraglutide provide substantial benefits, reducing diabetes risk by 79% and significantly delaying disease onset over 160 weeks [10]. As detailed in Table 5, these outcomes represent a paradigm shift toward glycemic remission as a feasible clinical goal. Achieving these targets is essential because sustained metabolic health is directly linked to the resolution of adiposity-related complications [10].

**Table 5:** Metabolic Remission Targets in Prediabetes and T2DM Description: Efficacy of high-potency incretins in achieving normoglycemia and preventing diabetes progression. Note: Normoglycemia is defined as HbA1c <5.7%. Tirzepatide shows the highest reported risk reduction for diabetes progression (93%). T2DM: type 2 diabetes mellitus. Source: Data from [10].

Medication	Outcome Metric	Result vs. Placebo
Tirzepatide	Progression to T2DM (176 weeks)	93% Risk Reduction
Semaglutide	Reversion to Normoglycemia (52 weeks)	81% of participants
Liraglutide	Risk of developing T2DM (160 weeks)	79% Risk Reduction

The therapeutic success of incretin-based therapies is supported by the relative preservation of GLP-1’s insulinotropic potency in diabetic patients, even when the physiological response to GIP is severely blunted [18]. While GIP mono-therapy often fails to lower glucose in hyperglycemic individuals, dual agonists like tirzepatide may partially restore GIP sensitivity by creating a biochemical liaison with GLP-1 [18]. This interaction enhances insulin secretion and glucagon suppression, facilitating the normalization of fasting glucose levels even in patients with long-standing diabetes [18]. Preclinical and mechanistic evidence suggests that biased receptor signaling and altered receptor recycling may contribute to the pharmacological effects of tirzepatide. However, the extent to which these mechanisms account for its superior clinical efficacy remains uncertain and requires further investigation [18]. Furthermore, restoring near-normal glucose levels through these potent therapies can create a virtuous cycle of improved glucose disposal and metabolic stability [10]. By leveraging these synergistic pathways, co-agonists achieve superior results in lowering HbA1c compared to traditional selective agents [18]. This shift toward complication-centric management aligns with the ABCD framework established in the introduction.

Cardiovascular disease

Establishing robust cardioprotective efficacy in secondary prevention is a central clinical priority for patients with established cardiovascular disease. Evidence from the landmark SELECT trial indicates that semaglutide 2.4 mg significantly reduces the risk of major adverse cardiovascular events (MACE) by 20% in adults with preexisting heart disease and overweight or obesity but without diabetes [3,19]. As shown in Figure 10, this statistically significant reduction highlights a consistent therapeutic effect across all obesity classes. The trial further demonstrated that semaglutide was associated with fewer serious adverse events, particularly those related to cardiovascular complications and infections [19]. The sustained weight loss of approximately 10.2% observed over a four-year period likely contributes to these long-term vascular benefits [19]. These findings support the use of once-weekly semaglutide 2.4 mg as a therapeutic option for cardiovascular risk reduction in adults with overweight or obesity and established cardiovascular disease without diabetes [19]. The framework proposed by McGowan et al. supports the integration of obesity pharmacotherapy into a complication-centered treatment approach that considers cardiovascular risk and established cardiovascular disease when selecting therapy [23]. These findings provide a strong foundation for integrating high-potency incretin mimetics into standard vascular care protocols to reduce the risk of cardiovascular death, myocardial infarction, and stroke.

**Figure 10:**
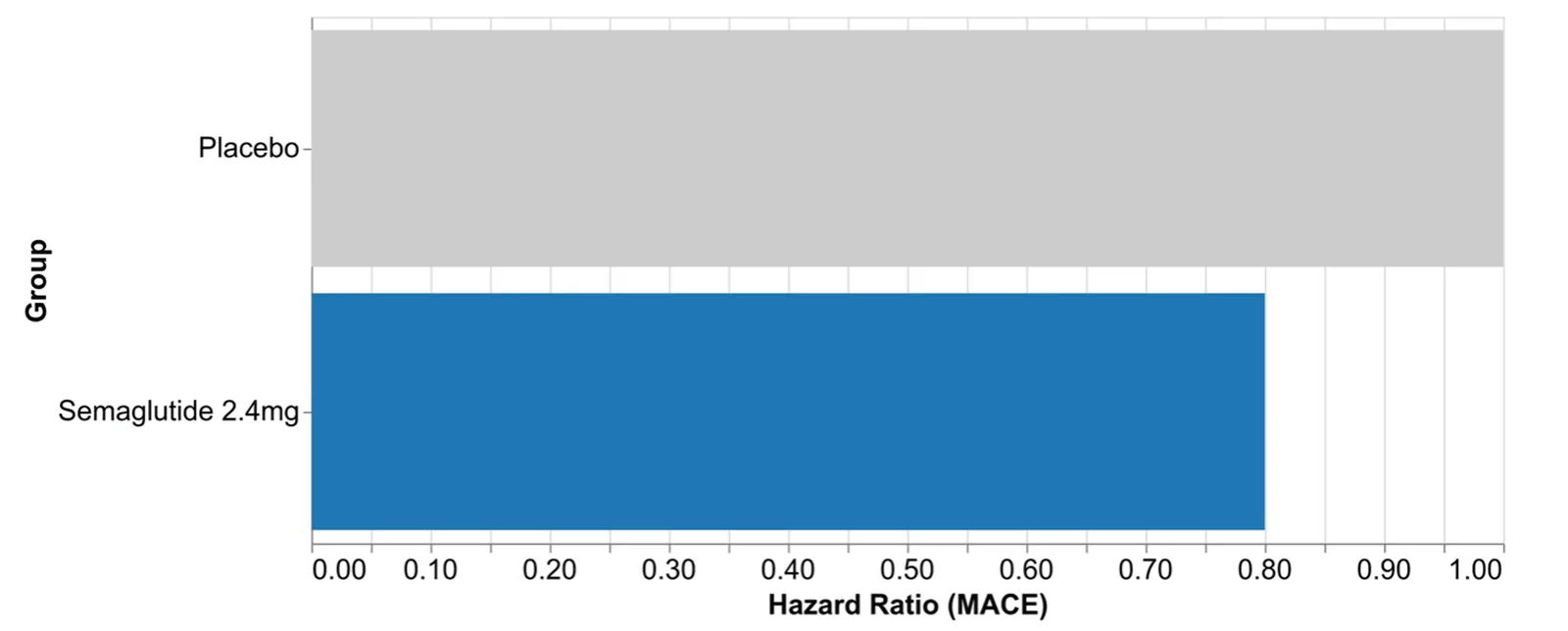
MACE Reduction in the SELECT Trial Description: Impact of semaglutide 2.4 mg on major adverse cardiovascular events (MACE) in non-diabetic patients. Note: MACE includes CV death, non-fatal MI, and non-fatal stroke. The 20% reduction was statistically significant (p < 0.001). CV: cardiovascular, MI: myocardial infarction. Source: Data from [3,19].

The management of obesity-related heart failure requires a nuanced approach that addresses specific hemodynamic challenges, such as plasma volume expansion and impaired vasodilation [3]. In patients with heart failure with preserved ejection fraction (HFpEF), semaglutide 2.4 mg appears to improve functional capacity, exercise tolerance, and quality of life, while significantly reducing systemic inflammation markers like high-sensitivity C-reactive protein (hsCRP) [3]. These clinical gains suggest that pharmacological weight loss is highly beneficial for the HFpEF phenotype, making it a primary therapeutic target. However, the European Society of Cardiology advises caution in patients with heart failure and reduced ejection fraction (HFrEF) to avoid exacerbating catabolic states that could worsen mortality outcomes [3]. By utilizing high-potency incretin therapies, clinicians can support the Adiposity-Based Chronic Disease (ABCD) framework, shifting the focus from simple body mass index reduction to the direct prevention and management of cardiac complications [3]. This complication-centric model emphasizes that metabolic stabilization and direct organ protection are the ultimate goals of modern obesity pharmacotherapy.

Metabolic dysfunction-associated steatotic liver disease (MASLD)

The hepatoprotective potential of selective GLP-1 receptor agonists is supported by a systematic review and meta-analysis including 27 trials, which indicates that these agents lead to a substantial reduction in liver fat content compared to control groups [24]. As shown in Table 6, this decrease in steatosis is accompanied by significant improvements in biochemical markers of liver health. Specifically, the pooled analysis reported effect estimates of -4.62 for alanine aminotransferase and -0.21 for aspartate aminotransferase [24]. Because the source article describes standardized mean differences in its Methods section, these estimates are presented without clinical units to avoid interpreting standardized effects as absolute changes in U/L. These benefits appear to be driven by a combination of direct modulation of hepatic lipid metabolism and indirect systemic effects, such as reduced visceral adiposity and waist circumference [24]. However, while early markers of injury and steatosis show robust improvement, evidence suggests that fibrosis-4 index (FIB-4) scores remain stable during typical trial durations [24]. This indicates that while GLP-1 receptor agonists effectively manage the early stages of metabolic liver disease, significant histological reversal of established fibrosis likely requires longer-term therapeutic intervention [24].

**Table 6:** Reduction of Liver Enzymes and Fat Content (MASLD) Description: Meta-analysis results for hepatic markers in patients treated with GLP-1 receptor agonists. Note: The source article describes the pooled hepatic estimates as “mean differences” in the Results section; however, its Methods section states that standardized mean differences were calculated for continuous outcomes. Accordingly, the estimates are presented here without clinical units to avoid interpreting standardized effects as absolute changes in U/L or percentage points. LFC: liver fat content, ALT: alanine aminotransferase, AST: aspartate aminotransferase, GGT: gamma-glutamyl transferase, MASLD: metabolic dysfunction-associated steatotic liver disease. Source: Data from [24].

Hepatic Marker	Effect Estimate	Significance (p-value)	Interpretation
ALT	-4.62	<0.001	Statistically significant reduction
AST	-0.21	<0.0001	Statistically significant reduction
GGT	-0.71	<0.0001	Statistically significant reduction
Liver Fat (LFC)	-1.38	<0.0001	Statistically significant reduction

The emergence of dual GIP/GLP-1 receptor agonists like tirzepatide suggests an even more potent pathway for addressing metabolic dysfunction-associated steatotic liver disease. Evidence from an MRI substudy indicates that tirzepatide may achieve superior efficacy in reducing liver fat compared to traditional metabolic interventions such as insulin degludec [13]. This high level of effectiveness likely stems from a synergistic dual mechanism that enhances insulin sensitivity and optimizes lipid profiles by reducing triglycerides and apolipoprotein B (apoB) levels [13]. Furthermore, tirzepatide appears to foster a holistic hepatoprotective environment by decreasing systemic inflammatory markers like C-reactive protein and ICAM-1 [13]. While direct activation of receptors in hepatocytes remains an unlikely primary driver, the systemic metabolic restoration achieved through dual agonism effectively targets the pathogenic mechanisms of fatty liver disease [13]. This shift toward multi-receptor targeting marks a significant advancement in the management of adiposity-based chronic diseases, positioning dual agonists as a central tool for preventing the progression of liver-related metabolic complications [13].

Chronic kidney disease

Establishing robust renal protection is a primary goal in the management of obesity-related metabolic dysfunction. A systematic meta-analysis of eight large-scale randomized controlled trials, encompassing over 60,000 patients, indicates that GLP-1 receptor agonists significantly reduce the risk of worsening kidney function [25]. Specifically, these agents were found to reduce the broader composite kidney outcome, which includes the development of macroalbuminuria, doubling of serum creatinine, or a significant decline in glomerular filtration, by approximately 21% [25]. Sensitivity analyses further suggest that these therapies lessen the risk of narrower outcomes, such as estimated glomerular filtration rate (eGFR) decline, by 18% [25]. These findings are supported by long-term cardiovascular trials like LEADER and SUSTAIN-6, which confirm sustained filtration rate preservation in treated populations [11]. The clinical relevance of this protection is underscored by a number needed to treat (NNT) of 47 over three years to prevent one major renal event [25]. Collectively, this evidence positions high-potency incretin mimetics as essential tools for slowing the progression of chronic kidney disease in high-risk metabolic cohorts.

The renoprotective benefits of GLP-1 receptor agonists stem from both direct intra-renal actions and systemic metabolic improvements. As illustrated in Figure 11, GLP-1 receptors are localized within the kidney to vascular smooth muscle cells of the afferent arterioles, where their activation promotes the acute excretion of sodium and water [11]. Beyond these hemodynamic effects, the paper by Hammoud and Drucker [11] points toward anti-inflammatory and anti-fibrotic actions, likely mediated by the inhibition of T-cell proliferation within renal tissues. While renal benefits are clear, Hammoud and Drucker [11] note that monitoring is required for rare instances of acute kidney injury secondary to severe gastrointestinal-induced dehydration. However, evidence indicates that human GLP-1 receptor agonists like liraglutide and semaglutide remain effective and safe for use in patients with significant renal impairment, including those with an eGFR as low as 25 mL/min per 1.73 m² [11,25]. This suggests that the gradual pathways of anti-inflammatory action and metabolic stabilization, including blood pressure and weight improvements, provide a robust protective framework even in advanced disease stages [25].

**Figure 11:**
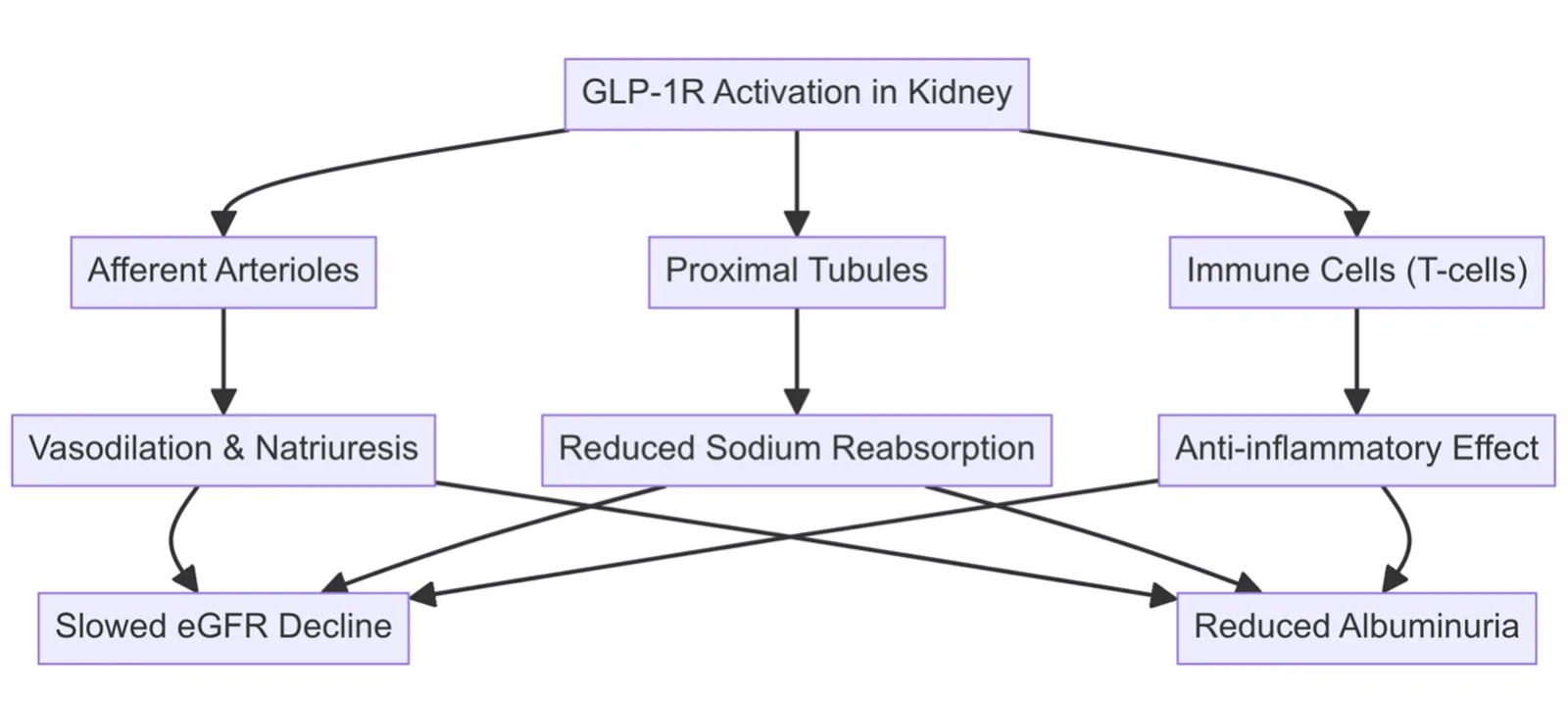
Renoprotective Actions of GLP-1 Receptor Agonists Description: Direct tubular and vascular effects of GLP-1 in the kidney leading to slowed CKD progression. Note: Natriuresis helps reduce fluid overload and blood pressure. Anti-fibrotic actions are mediated through T-cell inhibition in the renal tissue. GLP-1: glucagon-like peptide-1, GLP-1RA: glucagon-like peptide-1 receptor agonist, GLP-1R: glucagon-like peptide-1 receptor, eGFR: estimated glomerular filtration rate, CKD: chronic kidney disease. Source: Author’s own illustration based on [11,25].

Quality of life

Modern obesity management is transitioning toward a holistic framework that prioritizes mental well-being and social functioning alongside traditional weight loss goals [23]. This paradigm shift, often referred to as the Adiposity-Based Chronic Disease (ABCD) model, recognizes that high-potency agents like semaglutide and tirzepatide are particularly effective for resolving mechanical complications known as fat mass disease [23]. Clinical trial data indicates that tirzepatide treatment leads to significant improvements in physical function scores when measured by validated instruments such as the 36-Item Short Form Health Survey, version 2 (SF-36v2) and the Impact of Weight on Quality of Life-Lite Clinical Trials Version (WQOL-Lite-CT) [17]. As shown in Table 7, these functional gains appear to be dose-dependent, with the highest improvements consistently reported in the 15 mg dose groups [17]. By addressing the physical limitations imposed by excess fat mass, these therapies allow patients to regain mobility and participate more fully in daily activities, which represents a critical component of metabolic health restoration beyond simple glycemic control [17].

**Table 7:** Impact of Incretins on Quality of Life (QoL) Metrics Description: Improvements in physical and psychosocial functioning as measured by validated patient-reported outcome tools. Note: SF-36v2 measures general physical functioning. IWQOL-Lite-CT is a weight-specific quality of life instrument. SF-36v2: 36-Item Short Form Health Survey, version 2, IWQOL-Lite-CT: Impact of Weight on Quality of Life-Lite Clinical Trials Version, QoL: quality of life Source: Data from [17].

QoL Domain	Instrument	Result (Tirzepatide 15mg)	Significance (vs. Placebo)
Physical Function	SF-36v2	+3.8 Score Change	p < 0.0001
Weight-Related Function	IWQOL-Lite-CT	+15.2 Score Change	p < 0.0001
Psychosocial Wellbeing	IWQOL-Lite-CT	+14.2 Score Change	p < 0.0001

The clinical utility of dual GIP/GLP-1 agonists extends to specific mechanical conditions, with tirzepatide currently recommended as a first-line treatment for patients with obesity and obstructive sleep apnea (OSA) due to its ability to induce a clinically significant reduction in the apnea-hypopnea index [23]. Similarly, semaglutide appears to offer greater pain reduction in knee osteoarthritis compared to earlier agents like liraglutide, directly enhancing joint-related quality of life [23]. Beyond these physical mobility gains, weight reduction is associated with significant improvements in psychosocial composite scores, which likely reflect a reduction in obesity-related stress and social stigma [17]. Integrating these patient-specific goals and quality-of-life improvements represents a central value proposition for the long-term clinical management of obesity [23]. As summarized in Table 7, the cumulative evidence suggests that the ultimate success of incretin-based therapy is measured not only by kilograms lost but by the comprehensive improvement in the patient’s daily functional capacity and psychological wellbeing [17].

Future perspectives

The rapid clinical success of dual-receptor agonists marks a definitive transition in obesity management, moving from standard body weight reduction toward a future of precision multi-receptor targeting. This chapter examines the emerging innovation trajectory, beginning with an overview of the triple agonist era and the development of oral small-molecule breakthroughs that aim to overcome current administration barriers. By moving beyond universal treatment protocols, the field is shifting toward biomarker-driven and patient-centered models that prioritize individual metabolic phenotypes. The following sections provide a structured analysis of these emerging drug candidates, followed by a discussion on long-term research priorities, sustainability challenges, and the essential requirement for health equity in global obesity care.

Emerging incretin-based therapies (Retatrutide, CagriSema, MariTide, Amycretin)

The development of retatrutide represents a significant transition toward “triple-hormone-receptor agonist” strategies that target GIP, GLP-1, and glucagon receptors simultaneously to maximize metabolic impact [26]. Phase 2 results indicate a dose-dependent weight reduction that reached 24.2% in the 12 mg group at 48 weeks, suggesting a pharmacological potency limit that may approach the efficacy of bariatric surgery [26]. As shown in Figure 12, this triple-receptor approach currently holds the highest weight-loss potency among pipeline agents. The inclusion of glucagon agonism is hypothesized to enhance energy expenditure and substrate utilization beyond simple appetite suppression [26,27]. However, safety monitoring remains critical because triple agonists can increase heart rate by approximately 6.7 beats per minute, which may counteract some cardiovascular benefits [26,27]. These characteristics are summarized in Table 8.

**Figure 12:**
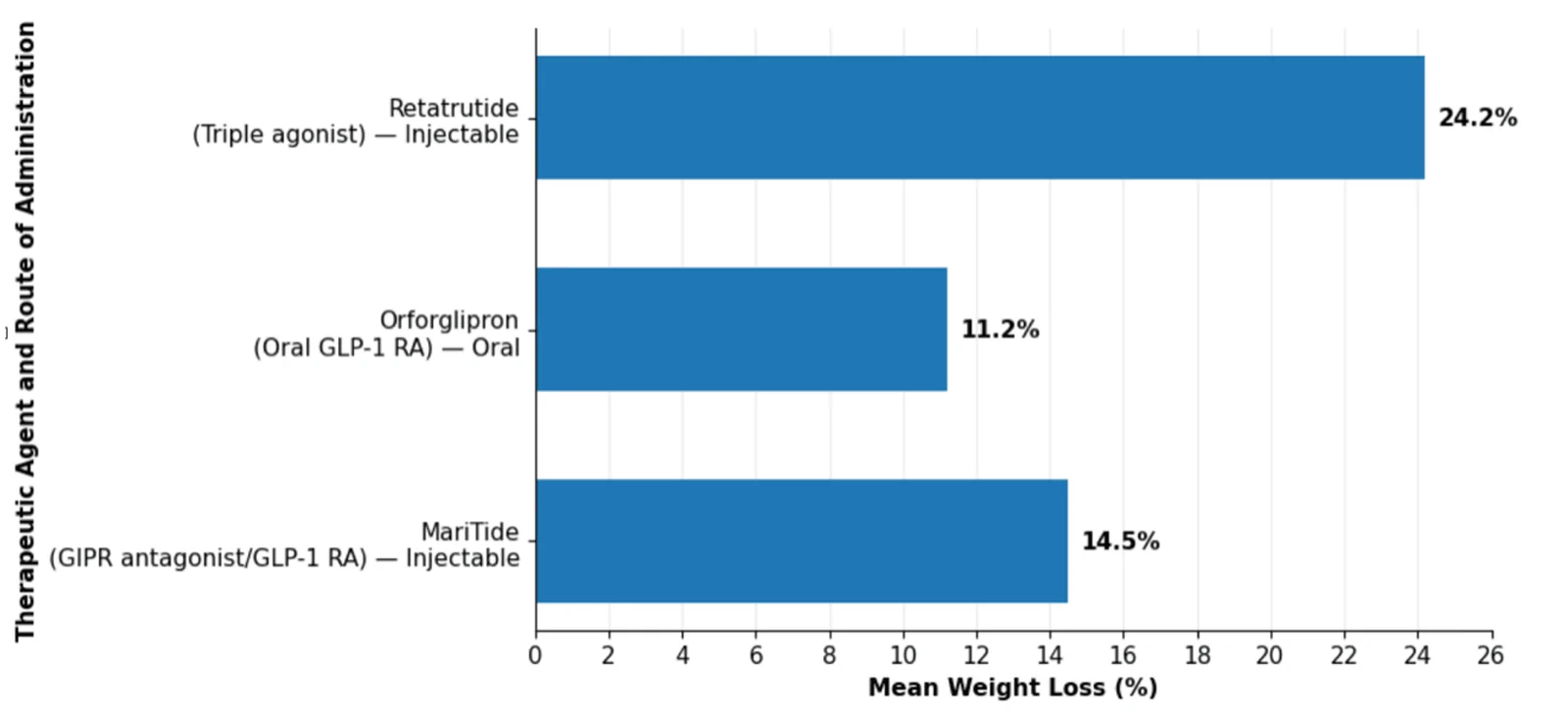
Comparative Weight Loss Efficacy of Pipeline Agents Description: Mean percentage body weight reduction across different pharmacological classes at primary trial endpoints. Note: Retatrutide data reflects the 12 mg dose at 48 weeks. Orforglipron data reflects the 36 mg dose at 72 weeks. MariTide (AMG 133) data reflects the 420 mg dose at day 85. GLP-1 RA: glucagon-like peptide-1 receptor agonist, GIPR: glucose-dependent insulinotropic polypeptide receptor. Source: Data from [26,28,29].

**Table 8:** Characteristics of Next-Generation Incretin Therapies Note: GIP: glucose-dependent insulinotropic polypeptide, GLP-1: glucagon-like peptide-1, SC: subcutaneous, GCG: glucagon receptor. Source: Based on [26-29].

Drug Name	Target Receptors	Route	Key Advantage
Retatrutide	GLP-1, GIP, GCG	Weekly SC	Highest weight loss potency
Orforglipron	GLP-1 (Small molecule)	Daily Oral	Patient preference, easier storage
MariTide	GLP-1 (Agonist), GIP (Antagonist)	Monthly SC	Extended half-life, durable effect
CagriSema	GLP-1, Amylin	Weekly SC	Synergistic metabolic control

Oral small-molecule agonists like orforglipron aim to overcome the storage and administration barriers associated with traditional peptide-based injections [28]. Clinical data from the ATTAIN-1 trial show that once-daily oral administration of 36 mg orforglipron achieved a mean weight loss of 11.2% over 72 weeks [28]. Notably, nearly 20% of participants on this highest dose achieved a weight reduction of at least 20%, which may be particularly relevant for patients with lower BMI values who constitute the majority of the population with excess adiposity [28]. This shift toward oral delivery is supported by strong patient preferences and the use of specialized absorption enhancers like sodium N-[8-(2-hydroxybenzoyl)amino]caprylate (salcaprozate sodium) (SNAC) to improve peptide bioavailability [27,28]. While orforglipron showed no signal of drug-induced liver injury, gastrointestinal events during the escalation phase remained the primary driver of treatment discontinuation [28]. The key features of these oral platforms are compared in Table 8.

MariTide (AMG 133) represents a novel engineering approach, utilizing a GIPR antagonist conjugated to GLP-1 analogs to enable a significantly extended terminal half-life of approximately 21 days [29]. Phase 1 data indicates that weight loss was maintained for up to 150 days after the last dose, potentially allowing for monthly or even less frequent dosing intervals [29]. As illustrated in Figure 12, MariTide demonstrates a unique efficacy profile even with less frequent administration. Other combination strategies, such as CagriSema, integrate GLP-1 receptor activation with amylin co-agonism to address the obesity-diabetes link more comprehensively [27]. These innovations reflect a broader move toward precision engineering and multi-receptor strategies intended to optimize long-term adherence and metabolic outcomes beyond what is achievable with current weekly mono-agonists [27]. A summary of these next-generation mechanisms is provided in Table 8.

Personalized medicine and future research

Individual variability in treatment response remains a major clinical hurdle, necessitating deeper research into the genetic and physiological determinants that influence how patients react to incretin-based therapies over time [30]. As the pharmacological landscape shifts toward complex dual- and triple-receptor agonists, future research will likely focus on discovering specific patient traits and biomarkers to predict individual reactions to these receptor combinations [30]. For instance, clinical evidence suggests that initial microbiotic profiles may serve as predictive phenotypes, offering a potential pathway for the development of microbiome-targeted adjuvant medicines [30]. Beyond improving efficacy, identifying responders through such biomarkers is proposed as a vital risk-stratified strategy to manage the high annual acquisition costs associated with modern obesity pharmacotherapy [30]. This transition toward precision medicine aims to replace universal protocols with tailored strategies based on individual metabolic characteristics.

A significant limitation of current high-potency therapies is the tendency for clinically significant weight regain following treatment discontinuation, which suggests that obesity requires chronic, long-term management strategies [21]. Currently, a critical research gap exists regarding the optimal duration and specific type of maintenance therapy needed to prevent this weight rebound [21]. As shown in Table 9, future research should move beyond simple assumptions regarding post-treatment weight trajectories to better characterize the complex biological processes underlying weight regain in real-world settings [4]. Furthermore, research is needed to evaluate the functional implications of the loss of lean mass and bone density that often accompanies rapid weight reduction [21]. Addressing these sustainability challenges is essential for shifting the therapeutic focus from short-term weight loss to permanent metabolic health and musculoskeletal stability.

**Table 9:** Current Research Gaps and Future Clinical Priorities Description: Key areas requiring further investigation to optimize obesity management. Note: These gaps represent the primary hurdles for moving from clinical trials to broad personalized medicine. Source: Based on [4,21,30].

Research Area	Specific Gap	Clinical Priority
Long-term Sustainability	Weight regain after cessation	Maintenance protocols
Body Composition	Loss of lean mass and bone density	Functional outcome studies
Safety	Rare events (e.g., thyroid, pancreatic)	Long-term surveillance databases
Health Equity	Underrepresentation of minority groups	Inclusive trial recruitment

The global application of modern obesity care faces substantial geographic disparities, as the majority of economic evaluations are currently focused on North America and Europe while missing data for high-prevalence regions like China [4]. Additionally, clinical trial populations frequently fail to reflect truly affected minority groups, which limits the generalizability of safety and efficacy data across different ethnicities [21]. Future research priorities must therefore include the integration of patient-centered perspectives and standardized quality-of-life metrics to ensure that the benefits of personalized medicine are equitable [4]. Furthermore, long-term safety surveillance for multi-receptor agonists, particularly regarding rare thyroid and pancreatic effects, remains a vital priority for establishing a robust evidence base in diverse populations [30]. Solving these equity and access barriers is critical for the worldwide implementation of the Adiposity-Based Chronic Disease framework.

Limitations

This review has several limitations that should be considered when interpreting its findings. First, although a structured literature search was conducted, the present study was designed as a narrative review rather than a systematic review. Consequently, a formal PRISMA-based study selection process and standardized risk-of-bias assessment were not performed. Second, the review was limited to English-language publications, which may have resulted in the omission of relevant evidence published in other languages. Third, considerable heterogeneity exists among the included studies in terms of patient populations, treatment duration, dosing regimens, comparators, and reported outcomes, which limits direct comparisons between individual incretin-based therapies. Furthermore, evidence regarding emerging agents such as retatrutide, CagriSema, MariTide, and amycretin remains comparatively limited, with several compounds still undergoing clinical development. Long-term data regarding treatment durability, safety, adherence, and weight regain following treatment discontinuation also remain incomplete. Finally, incretin-based obesity pharmacotherapy is a rapidly evolving field, and additional clinical trial results may modify the current understanding of the comparative efficacy and safety of these therapies. Therefore, conclusions regarding newer agents should be interpreted in the context of the evidence available at the time of this review.

## Conclusions

Modern obesity management is undergoing a fundamental shift away from body mass index as the sole measure of success, moving instead toward the complication-centric model of Adiposity-Based Chronic Disease. This framework prioritizes the pathophysiology of adipose tissue and its impact on systemic health over body weight alone. The emergence of second-generation medications, specifically tirzepatide and semaglutide, provides the pharmacological potency required to fulfill this model. These agents achieve a predictable clinical response by reaching weight-loss thresholds of 15% or more, which are essential to prevent or improve a broad array of obesity-related complications. Achieving such targets allows for the potential remission of severe conditions like type 2 diabetes and obstructive sleep apnea. Ultimately, long-term success requires recognizing obesity as a complex neurohormonal condition that necessitates a lifelong, individualized therapeutic roadmap rather than a series of short-term interventions. This approach ensures that treatment intensity is matched to the severity of organ dysfunction and metabolic risk. The long-term clinical benefits of incretin-based therapies are likely to depend in part on sustained treatment, as weight regain is common following treatment discontinuation. Long-term management should therefore prioritize treatment tolerability, adherence, and individualized strategies that support the maintenance of metabolic and cardiovascular benefits achieved during active therapy. Further evidence is needed to define optimal treatment duration and maintenance strategies, particularly for newer multi-receptor therapies.
